# Utility of a Novel Biofeedback Device for Within-Breath Modulation of Heart Rate in Rats: A Quantitative Comparison of Vagus Nerve vs. Right Atrial Pacing

**DOI:** 10.3389/fphys.2016.00027

**Published:** 2016-02-04

**Authors:** Erin L. O'Callaghan, Ashok S. Chauhan, Le Zhao, Renata M. Lataro, Helio C. Salgado, Alain Nogaret, Julian F. R. Paton

**Affiliations:** ^1^School of Physiology, Pharmacology and Neuroscience, University of BristolBristol, UK; ^2^Department of Physics, University of BathBath, UK; ^3^Department of Physiology, School of Medicine of Ribeirão Preto, University of São PauloSão Paulo, Brazil

**Keywords:** respiratory sinus arrhythmia, novel biofeedback device, cardiac pacing, vagal nerve stimulation, heart rate variability

## Abstract

In an emerging bioelectronics era, there is a clinical need for physiological devices incorporating biofeedback that permits natural and demand-dependent control in real time. Here, we describe a novel device termed a central pattern generator (CPG) that uses cutting edge analog circuitry producing temporally controlled, electrical stimulus outputs based on the real time integration of physiological feedback. Motivated by the fact that respiratory sinus arrhythmia (RSA), which is the cyclical changes in heart rate every breath, is an essential component of heart rate variability (HRV) (an indicator of cardiac health), we have explored the versatility and efficiency of the CPG for producing respiratory modulation of heart rate in anesthetized, spontaneously breathing rats. Diaphragmatic electromyographic activity was used as the input to the device and its output connected to either the right cervical vagus nerve or the right atrium for pacing heart rate. We found that the CPG could induce respiratory related heart rate modulation that closely mimicked RSA. Whether connected to the vagus nerve or right atrium, the versatility of the device was demonstrated by permitting: (i) heart rate modulation in any phase of the respiratory cycle, (ii) control of the magnitude of heart rate modulation, and (iii) instant adaptation to changes in respiratory frequency. Vagal nerve pacing was only possible following transection of the nerve limiting its effective use chronically. Pacing via the right atrium permitted better flexibility and control of heart rate above its intrinsic level. This investigation now lays the foundation for future studies using this biofeedback technology permitting closer analysis of both the function and dysfunction of RSA.

## Introduction

Respiratory sinus arrhythmia (RSA) is the physiological phenomenon whereby heart accelerates during inspiration and decelerates during expiration (Anrep et al., 1936). Whilst the physiological functions of RSA remain ambiguous (Hayano et al., 1996; Ben-Tal et al., 2012; Elstad et al., 2015), it is clear that the absence of RSA is a strong prognostic indicator of cardiovascular pathology (La Rovere et al., 1998). Thus, increasing heart rate variability (HRV) by reinstating RSA may improve outcome in patients with cardiovascular diseases such as heart failure.

Cyclic modulation of heart rate (HR) within each breath is not possible with existing medical device technology. Such technology must be a closed-loop system that generates a physiologically-appropriate stimulus in response to a biological input, such as respiration, in real time. Therefore, we have developed a novel analog-hardware central pattern generator (hCPG) that integrates Hodgkin-Huxley equations in real time to recapitulate the outputs and dynamics of neurons. The advantage of hCPG neurons is that their firing sequence is extremely stable but both highly sensitive and fast to respond (microseconds) to changes in input signal. By adapting the hCPG neuron phase lags to changes in rhythm frequency, the hCPG can maintain precise timing of burst discharges on a breath by breath basis (Nogaret et al., 2013; Zhao and Nogaret, 2015) that can be used to pace the heart. Functional circuits have been simulated (Briggman and Kristan, 2008) but it this the first time their principles are being implemented in hardware for the application of neurostimulation *in vivo*.

We have previously demonstrated the proof-of-principle hCPG in an *in situ* rat preparation (Nogaret et al., 2013). This device was optimized to stimulate the vagus nerve (termed the VN-CPG) and elicited periodic bradycardia at different times within the respiratory cycle. The resulting HR waveform is referred to as respiratory-modulated heart rate (RMHR). The parameter space and biofeedback capabilities were neither fully explored nor quantified, which is now a critical step in the pathway to translation of the device. To fully exploit this device's potential for translation we sought to investigate its ability to generate RMHR as a cardiac pacemaker. Thus, in the present study, we aimed to: (a) assess the relationship between the dynamic respiratory input signal of an intact rat and the range of effective output of the hCPG, (b) explore and quantify the flexibility of vagal induced respiratory modulation of heart rate, (c) convert the hCPG into a direct cardiac pacemaker (which will be referred to as the PM-CPG) and explore its feasibility and potential for respiratory modulated (i.e., breath by breath) naturalistic cardiac pacing.

## Methods

### Ethical approval

All animal experiments were performed in accordance with institutional guidelines and the UK Home Office (Scientific Procedures) Act (1986) with project approval from the University of Bristol, University ethics of research committee.

### Surgical preparation

Male Wistar rats (250–350 g, *n* = 8) were anesthetized with isofluorane (induction 5%, maintenance 1.5–1.8% in 100% oxygen). Depth of anesthesia was monitored regularly and deemed adequate by an absence of pedal withdrawal and corneal reflex responses. Core temperature was monitored and maintained (37.1–37.6°C) by homeothermic blanket (Harvard Apparatus Ltd, UK). Rats were instrumented to record arterial pressure, continuous diaphragmatic electromyogram (dEMG) and HR as follows. Fluid-filled polyethylene catheters were inserted into the right carotid artery (0.9% saline, 100 IU/mL heparin) and right jugular vein to record arterial pressure and maintain hydration (~3 mL/kg/hr of 0.9% saline) respectively. A pair of nylon-insulated stainless steel wire electrodes (0.25 mm insulated diameter) ending with a suture pad (0.7 × 1.0 × 3.2 mm, Plastics1, USA) were placed 3–4 mm apart onto the costal diaphragm, accessed via medial abdominal incision, for continuous dEMG (contracts only during inspiration) recordings. The electrode wires were tunneled out of the abdomen and connected to the amplifier (AM systems, USA). Another pair of subcutaneous electrodes to capture the electrocardiogram R wave were secured bilaterally to the external intercostal muscles between the second and fourth ribs.

#### Vagal nerve electrode placement

**Figure 1:**
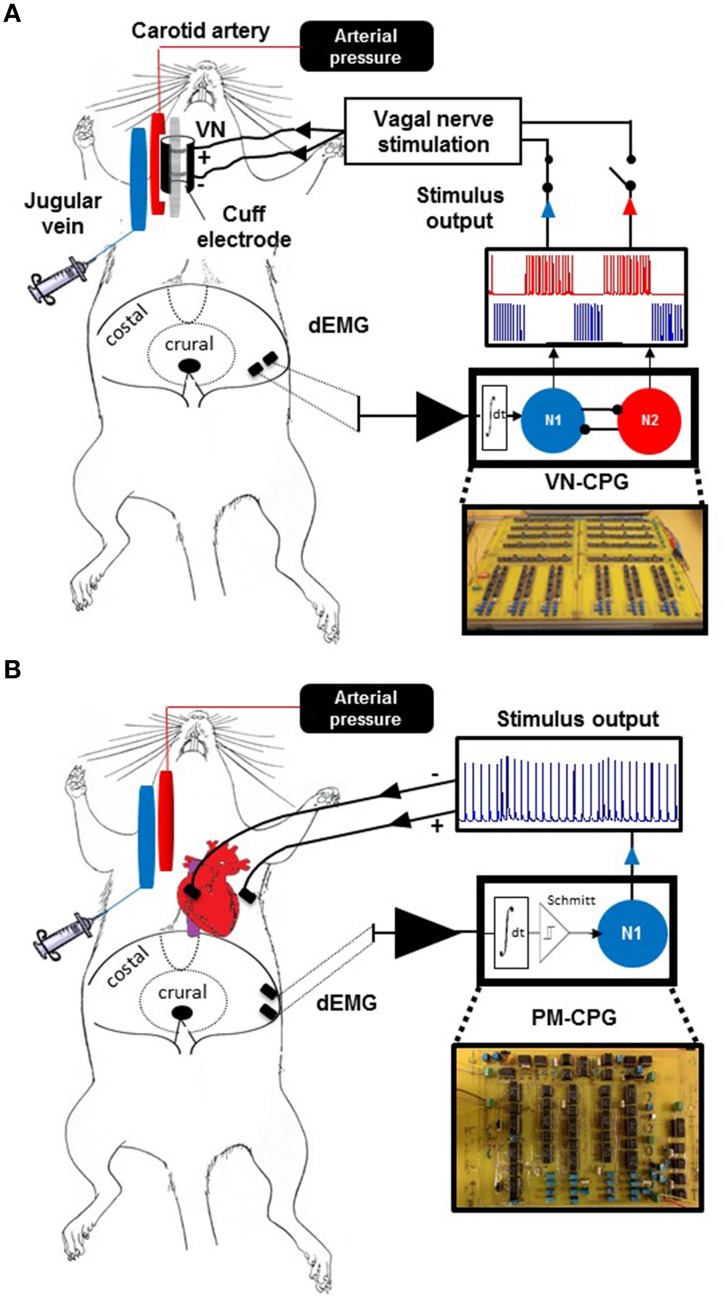
**Biological interface of the VN-CPG and PM-CPG**. Experimental setup in which the VN-CPG receives input from dEMG **(A)**. A view of the abdominal surface of the diaphragm shows the relative placement of two electrodes within the costal diaphragm which are used to record dEMG without interference from cardiac electrical activity. The black circle within the crural diaphragm indicates the oesophageal path. The dEMG activity is amplified and filtered by a pre-amplifier (black triangle) before use as the respiratory input to the VN-CPG. The VN-CPG is shown (bottom right) and a simplified representation of the differential amplifier and inhibitory interneurons, N1 (blue) and N2 (red), that comprise the VN-CPG (detailed in Zhao and Nogaret, 2015) and their respective firing patterns are depicted above. Either N1 or N2 can be connected to the bipolar cuff electrode enclosing the right cervical vagus nerve to control HR. **(B)** The dEMG signal was again used as a respiratory input to the PM-CPG. The signal forming stages are indicated as above, with the addition of a Schmitt trigger differential amplifier. The signal is applied to N1, which generates continuous output with inspiratory modulation of spike frequency (blue trace, inset). The output was used to pace the heart using a myocardial bipolar electrode. dEMG, diaphragm electromyogram; N1, neuron 1; N2, neuron 2; PM-CPG; hCPG format for use as a cardiac pacemaker; VN-CPG, hCPG format for vagal nerve stimulation.

The right cervical vagus nerve was accessed by midline incision, separated from the common carotid artery using a glass hook and placed inside a bipolar cuff electrode, which was closed with a suture (Polyester braided 6-0, Ethicon, USA). The voltage-based stimulus output of the VN-CPG was directly connected to the bipolar cuff electrode leads (Figure 1A). Unilateral vagotomy was performed by tying two sutures around the nerve cranial to the cuff electrode and transecting the nerve between the sutures.

#### Cardiac lead placement

In separate rats, bipolar cardiac pacing was conducted using two nylon-insulated stainless steel leads ending a suture pad as electrodes. The rat was ventilated (2.5 mL/breath, 85 breaths per min) and a right lateral thoracotomy was made in the 3rd intercostal space. The ribs were retracted and pericardium ruptured to access and view the right atrium and adjacent myocardium. A cotton swab was used to physically stabilize the heart whilst one electrode pad (depolarizing lead) was sutured to the myocardium in close proximity to the right atrium and the other placed within the muscles of the 3rd intercostal space. The chest was closed and negative intra-thoracic pressure reinstated by suction. Once spontaneous breathing resumed, the electrode leads were connected to the voltage-based stimulus output of the PM-CPG as shown in Figure 1B.

### Design of the vagus nerve (VN)-CPG

A comprehensive description of the design and functionality of the VN-CPG has been published previously (Zhao and Nogaret, 2015) and is briefly summarized here. The VN-CPG comprises two spiking neurons 1 and 2 (N1 and N2 respectively) interconnected through mutually inhibitory links. Each link consists of a differential transconductance amplifier which injects a post-synaptic current proportional to the voltage difference between the two neurons *I*_1 → 2_ = *g*(*V*_2_ − *V*_1_). Mutual inhibition causes the two neurons to oscillate out of phase. Therefore, when the dEMG signal is applied to N1, N1 oscillates over the whole duration of the inspiratory phase whilst N2 remains silent (Figure 1A, blue). N2 begins to oscillate as soon as inhibition from N1 is released. A time delay (τ) was introduced via a RC circuit to extend the duration of signals from N1 and slow the rise of the current stimulating N2. This enabled fine-tuning of N2 burst duration, such that N2 was firing during the latter half of expiration (Figure 1A, red). Each neuron may be viewed as an analog computer which integrates the Hodgkin-Huxley equations in real time. The circuit models the voltage gated conductance of the sodium, potassium, calcium, and the leakage channels using VLSI circuits (Mahowald and Douglas, 1991; Nogaret et al., 2013). These were implemented using discrete current mirror components (Advanced Linear Devices) on printed circuit boards as we described previously (Nogaret et al., 2013). The dEMG signal consists of 50–100 μV bipolar bursts. This raw signal was pre-amplified (× 10^4^ gain) and band pass filtered (300–500 Hz; AM systems, USA) before processing by a series of signal forming stages built on the printed hCPG circuit board to obtain clean current pulses delimiting the inspiratory phase. These signal forming stages included a fine amplifier (× 20 gain), a rectifier stage, a controllable delay stage allowing the duration of N1 oscillations to overlap with the expiratory phase as desired (see below), a two stage low pass Butterworth filter, and a transconductance amplifier converting the filtered/rectified dEMG signal into current pulses for stimulating the hCPG. The output stage included an impedance matching stage to lower the output impedance of the device and allow tuning the amplitude of neuron spikes (0–4 V tuneable range). We have performed preliminary trials of synchronization to respiration by stimulating the hCPG with recordings of dEMG through a NI6259 DAQ card from National Instruments.

The tuneable intra-burst stimulus parameters were intra-burst frequency (from 1 to >500 Hz) and amplitude (0–3.3 V using 8 V source input). Individual spikes approximated a triangular shape and were 2 ms wide at the base. The intra-burst frequency can be directly controlled and increased above 500 Hz.

#### Conversion to a direct pacing (PM) CPG

**Figure 2:**
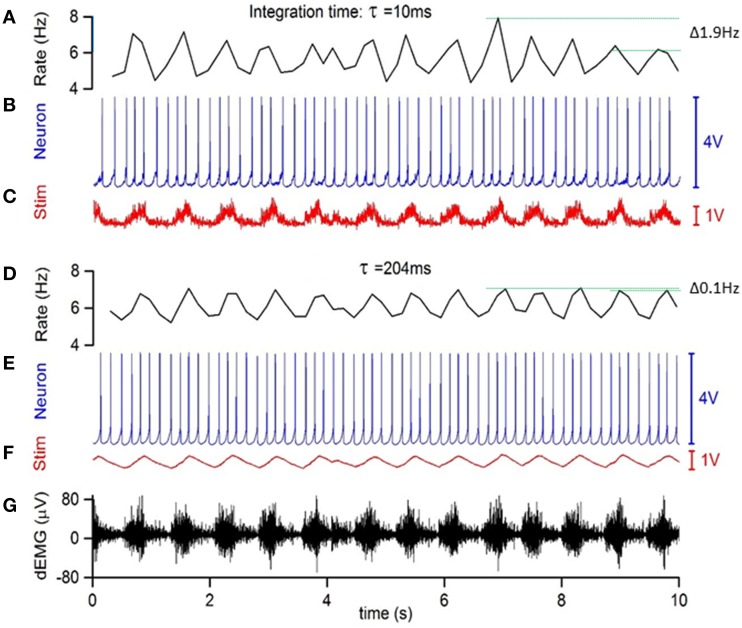
**Low pass filtering of simulated respiratory input on the accuracy of instantaneous frequency modulation. (A)** Inverse inter-spike interval of **(B)** N1 stimulated by current modulation **(C)**. This current modulation was obtained using a 10 ms integration time to integrate the raw dEMG input **(G)**. The low frequency variations in inter-spike interval reached 1.9 Hz, indicated by horizontal green lines. **(D–F)** same as above but using a 204 ms integration time to obtain the frequency modulation, which reduced low frequency variations in inter-spike-interval to 0.1 Hz.

Inducing RMHR through direct right atrial stimulation required accurate control of the inter-spike interval. This is particularly important during the transition between inspiratory and expiratory periods. The accurate modulation of inter-pulse intervals (and therefore the instantaneous HR) was achieved using the excitatory response of neuron N1 to modulate the frequency of sinoatrial stimulation. This approach required the complete elimination of noise from the rectified dEMG signal input to N1. This was achieved by additional signal forming stages including Butterworth filters, a Schmitt trigger and a differential amplifier (Figure 1B) to control the base HR level and RMHR independently. The effect of smoothing dEMG by increasing the integration time of the Butterworth filter from 10 to the 204 ms used in this study is shown in Figure 2.

### Experimental protocol

#### Respiratory modulation of HR using the VN-CPG

A voltage dose-HR response curve was generated for each rat (*n* = 4) by measuring the mean HR after unilateral vagotomy, before and during tonic vagal nerve stimulation (20 Hz) starting at 0.5 V and increasing in 0.25 V increments until the bradycardia reached a plateau. The voltage at which a half-maximal HR response was produced is termed V_50_. The 20 Hz frequency was selected as it gave the most distinct stimulus voltage-HR response curve in pilot experiments and is within the physiological range (McAllen et al., 2011). RMHR amplitude was recorded during N1- and N2- based stimulation (i.e., in phase with inspiration or expiration, respectively) at 5, 10, 20, 30, and 40 Hz intra-burst frequencies. Average burst duration was matched for N1 and N2 and each was arbitrarily set to ~36% of the respiratory cycle length for the above protocols while stimulus amplitude was V_50_. We measured RMHR amplitude in response to increasing burst duration (0.2 s increments from 0.2 to 0.8 s duration) of N2 only, at 20 Hz intra-burst frequency and V_50_.

#### Respiratory modulation of HR using the PM-CPG

To define the physiological working range of heart rates for PM-CPG, a maximum and minimum HR was experimentally determined in each rat (*n* = 4). The maximum HR was found by metronomic pacing, starting at 450 bpm and increasing in 20 bpm increments until an ECG R wave did not produce a blood pressure peak. This indicated ineffective ventricular filling and/or contraction. The minimum HR was defined as the HR 10 min after atenolol (1 mg/kg) was administered intravenously. Within these pre-determined minimum and maximum HR boundaries, rats were paced using the PM-CPG at RMHR amplitudes of 20, 40, and 60 bpm from the minimum HR upwards to the maximum HR in 50 bpm increments.

### Statistical analysis

Data were analyzed using GraphPad Prism (v5). All grouped data are mean ± SEM and a statistically significant difference was reported when *P* < 0.05. Two-way ANOVA followed by Tukey's multiple comparison test was used to compare the effect of VN-CPG stimulus phase (N1 and N2) and stimulus amplitude or frequency on the amplitude of RMHR. One-way ANOVA and Tukey's multiple comparisons test was used to compare the effect of RMHR generated by VN-CPG on mean HR. A one sample *t*-test was used to determine if a HR waveform had significantly phase shifted from its reference value.

## Results

### Instantaneous processing of biological respiratory input signals by the VN-CPG

**Figure 3:**
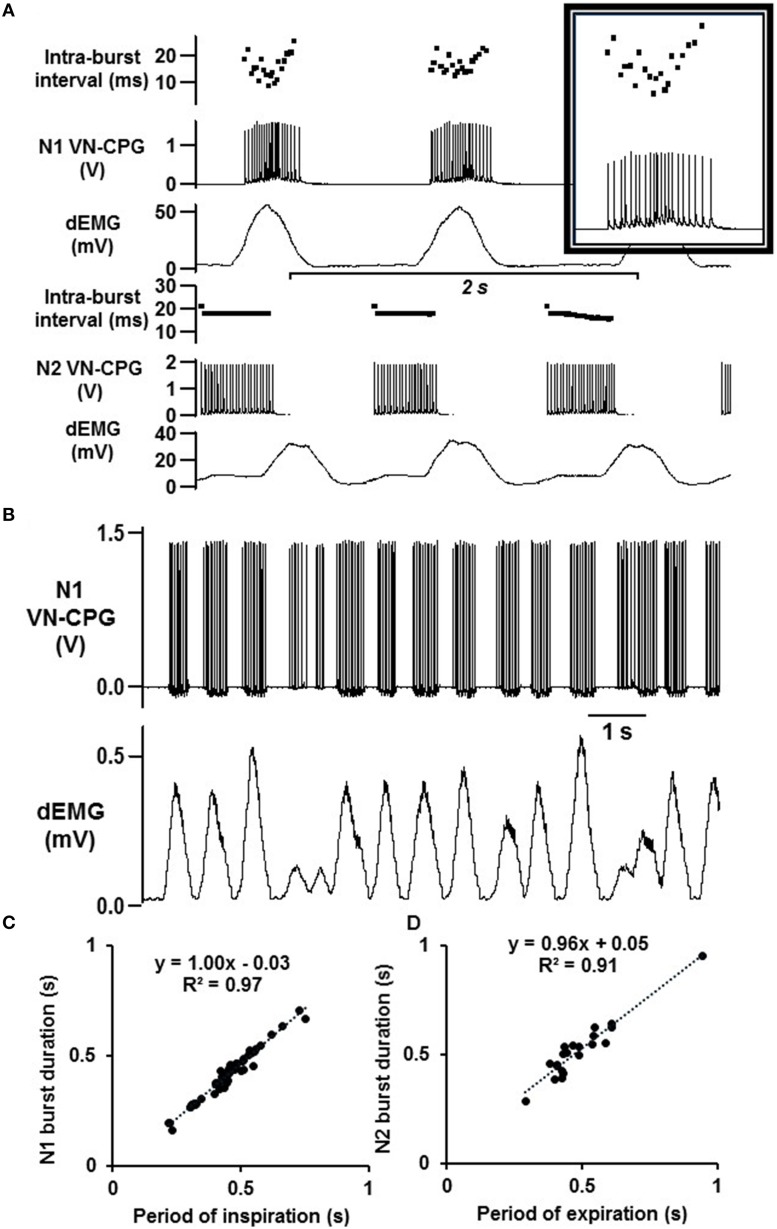
**Instantaneous VN-CPG processing enables biological feedback. (A)** The amplitude and intra-burst interval of the VN-CPG N1 mimics the dEMG input signal, where the N1 amplitude and intra-burst interval (inset) fluctuate with the height of the dEMG amplitude. N2 is inhibited when N1 activity reaches a threshold and therefore exhibits a constant amplitude and intra-burst interval during the expiratory period. The timing of N1 bursts instantaneously adapts to spontaneous changes in breathing **(B)**. The duration of N1 **(C)** and N2 **(D)** are directly related to the periods of inspiration and expiration respectively. The slope of the linear regression for N1 is 1.00 (*R*^2^ = 0.97), indicating the instantaneous processing of dEMG input and conversion into N1 stimulus output to precisely coincide with inspiration. dEMG, diaphragm electromyogram; VN-CPG, hCPG for vagal nerve stimulation.

All experiments using the VN-CPG were conducted under anesthesia during spontaneous breathing (67 ± 10 breaths per min, *n* = 4). The gain of the VN-CPG input was finely adjusted (up to 20 ×) to tune the dEMG signal of each rat such that two distinct bursting phases were generated from the VN-CPG outputs N1 and N2 (Figure 3A). The pattern of N1 bursts reflect precisely, and in real time, the pattern of dEMG input signal (Figure 3A), highlighting the instantaneous processing capabilities of the VN-CPG. That is, N1 intra-burst interval is shortest during peak dEMG activity (Figure 3A inset). This pattern is not observed for N2 bursting because it is not integrating the dEMG input.

The VN-CPG outputs responded instantly to dynamic breathing and a representative example in Figure 3B highlights the N1 burst duration being modulated by changes in dEMG amplitude, frequency, and width. The accuracy of the VN-CPG is evident from the linear relationship between N1 burst duration and the period of inspiration (slope = 1.00, *R*^2^ = 0.97) and between N2 burst duration and the period of expiration (slope = 0.96, *R*^2^ = 0.91) over a range of respiratory intervals (RI) (Figures 3C,D).

### The VN-CPG controls the phase of the RMHR waveform

**Figure 4:**
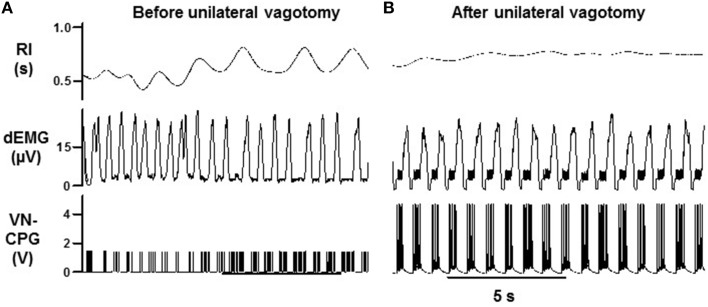
**Breathing stabilizes during VN-CPG stimulus after unilateral vagal nerve transection**. The respiratory interval changed erratically during VN-CPG stimulation of the vagus nerve **(A)**. This variation was prevented by transecting the vagus nerve cranial to the stimulating electrodes, thereby blocking afferent signal transduction **(B)**. dEMG, diaphragmatic electromyogram; RI, respiratory interval; VN-CPG, hCPG format for vagal nerve stimulation.

**Figure 5:**
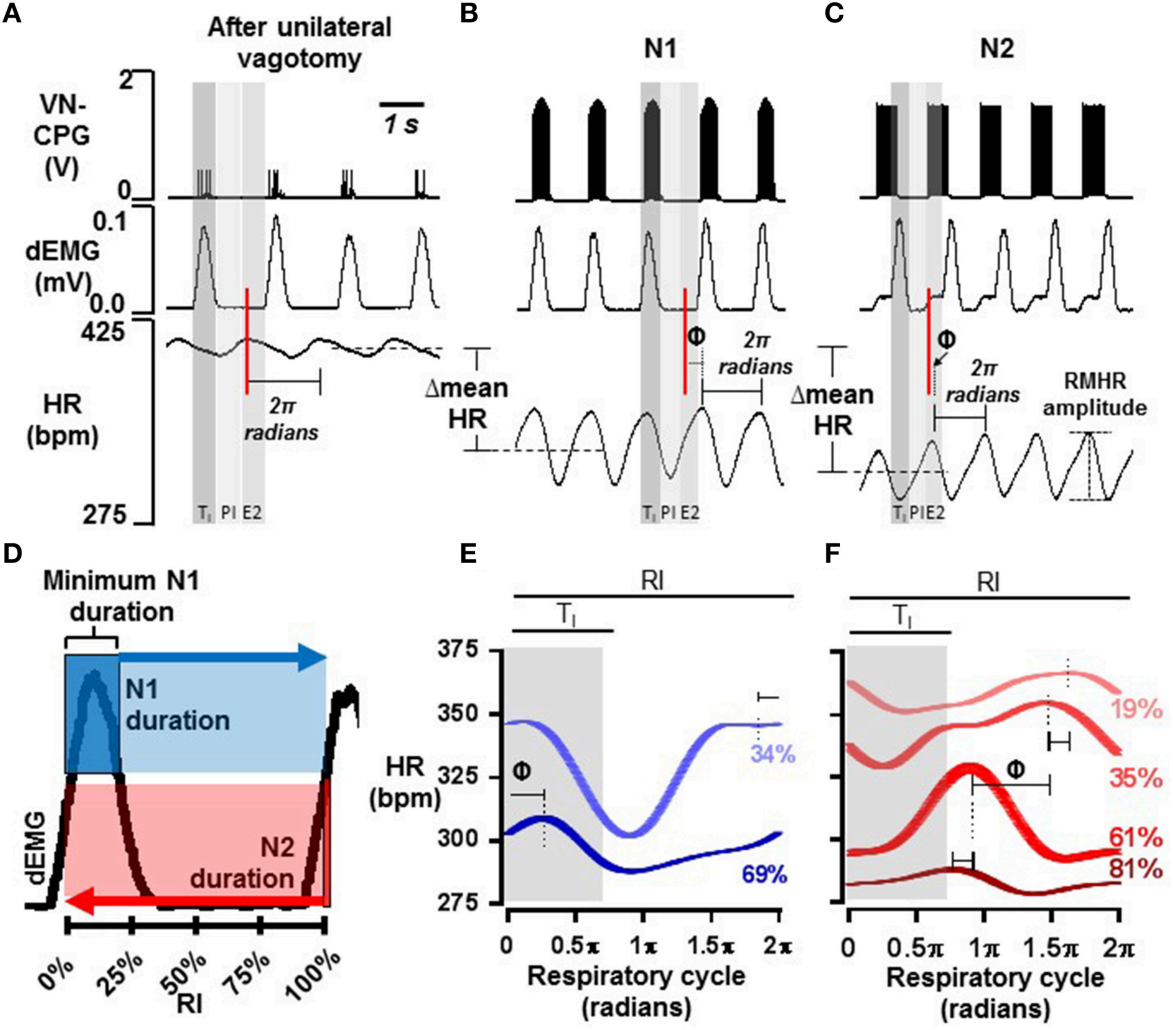
**RMHR generated by the VN-CPG: phase control**. Representative examples of RSA after unilateral vagotomy **(A)** and during VN-CPG stimulation from either N1 **(B)** or N2 **(C)** outputs. Shaded areas indicate the arbitrary division of the respiratory cycle into thirds: inspiration (T_I_), post-inspiration (PI), and late expiration (E2). An example of the HR waveform over one respiratory cycle (2π radians in length) is indicated on panels **(A–C)**. The peak HR is used to align the start of the HR waveform and this reference from panel **(A)** is shown as a red line. The shift of the HR waveform during N1- and N2- VN-CPG stimulation (panels **B,C** respectively) from that in panel **(A)** is denoted by Φ. The decrease in mean HR that occurred during VN-CPG stimulation from both outputs is also shown. **(D)** Minimum N1 burst duration as a proportion of the respiratory interval (RI) is indicated in dark blue; its duration could be increased to the full duration of the respiratory cycle. N2 burst duration (red), which results from the inhibitory influence of N1, could be as short as 1 spike and as long as the duration of the respiratory cycle. Panels **(E,F)** show representative examples of HR waveforms from one animal where multiple N1 (blue, **E**) and N2 (red, **F**) stimulus burst durations were applied. HR waveforms were averaged over 60 respiratory cycles, where one respiratory cycle was normalized to 2π radians. Stimulus burst durations are expressed as a percentage of the respiratory interval and color-coded to match the resultant HR waveforms. The duration of dEMG burst activity (T_*I*_) is shaded gray and Φ between two waveforms is also indicated. 2π radians, standardized respiratory cycle period; Φ, phase shift; dEMG, diaphragm electromyogram; E2, late expiration; HR, heart rate; N1, neuron 1; N2, neuron 2; PI, post-inspiration; RMHR, respiratory-modulated heart rate; T_I_, period of inspiration; RI, respiratory interval; VN-CPG, hCPG for vagal nerve stimulation.

The baseline HR waveform in anesthetized rats was associated with respiration, where peak HR occurred during mid-late expiration and decreased over inspiration. Different RSA patterns have been observed in rats and this is reviewed in the discussion. The peak-trough magnitude of this RSA was 11 ± 2 bpm (*n* = 4). Upon connecting the VN-CPG to stimulate the vagus nerve, spontaneous breathing became erratic (Figure 4A). Transection of the vagus nerve cranial to the stimulating electrodes prevented the unwanted respiratory variability caused by afferent vagal nerve fiber activation (Figure 4B) and was routinely conducted for subsequent experiments using the VN-CPG. RSA was 7 ± 2 bpm (*n* = 4) after unilateral vagotomy and the pattern, where HR decreased during inspiration, was unchanged (Figure 5A).

The HR waveform averaged over multiple breaths (at the same respiratory rate) approximated a sine wave, thus we could quantify a phase shift (Φ) in the HR waveform generated by the different VN-CPG stimulus modalities. The respiratory cycle was normalized to 2π radians and the occurrence of peak HR within the respiratory cycle was used as a reference point for calculating the phase shift. An example of a reference peak in HR during RSA after unilateral vagotomy is indicated by the red line in Figure 5A and the phase shift from this point during VN-CPG stimulation via N1 and N2 is indicated by Φ in Figures 5B,C respectively.

RMHR was induced by connecting either N1 (burst duration 26 ± 2% RI) or N2 (burst duration 31 ± 3% RI) outputs of the VN-CPG to the right vagus nerve after unilateral nerve transection cranial to the stimulating electrode. Connecting N1 caused a robust, transient bradycardia during late-inspiration to mid-expiration that tended to be shifted 0.25 ± 0.08π radians (*n* = 4, *P* = 0.06) rightward compared to the unstimulated HR waveform (Figure 5B). Connecting N2 generated a similarly robust and transient bradycardia which was not significantly shifted (0.19 ± 0.18π radians, *n* = 4, *P* = 0.40) from the unstimulated HR waveform but was 0.31 ± 0.04π radians (*n* = 4, *P* = 0.003) shifted leftward from the RMHR generated by N1 (Figure 5C, N2).

N1 and N2 burst durations could be varied continuously by tuning the decay time of slowly inactivating currents as indicated in Figure 5D. Minimum N1 burst duration was 66 ± 2% of the inspiratory period (T_I_) during slow breathing (1.0 s respiratory interval) where T_I_ is approximated from dEMG activity. N1 burst duration could be extended to the full respiratory interval. Minimum N2 duration was 1 spike and could also be extended to encompass the full respiratory interval, but from the opposite direction to N1 (Figure 5D). An increase in N1 burst duration from 31 ± 1 to 57 ± 4% of the respiratory interval shifted peak HR 0.22 ± 0.05π radians (*n* = 4, *P* = 0.02) from late expiration to early inspiration of the following breath. A representative example is shown in Figure 5E.

Increasing N2 burst duration from 31 ± 3 to 73 ± 6% of RI shifted the peak HR earlier in the respiratory cycle (Φ = −0.44 ± 0.11π radians, *n* = 4, *P* = 0.03). A representative animal shown in Figure 5F indicates the variation on RMHR pattern with increasing N2 burst duration.

### Amplitude of RMHR generated using the VN-CPG

**Figure 6:**
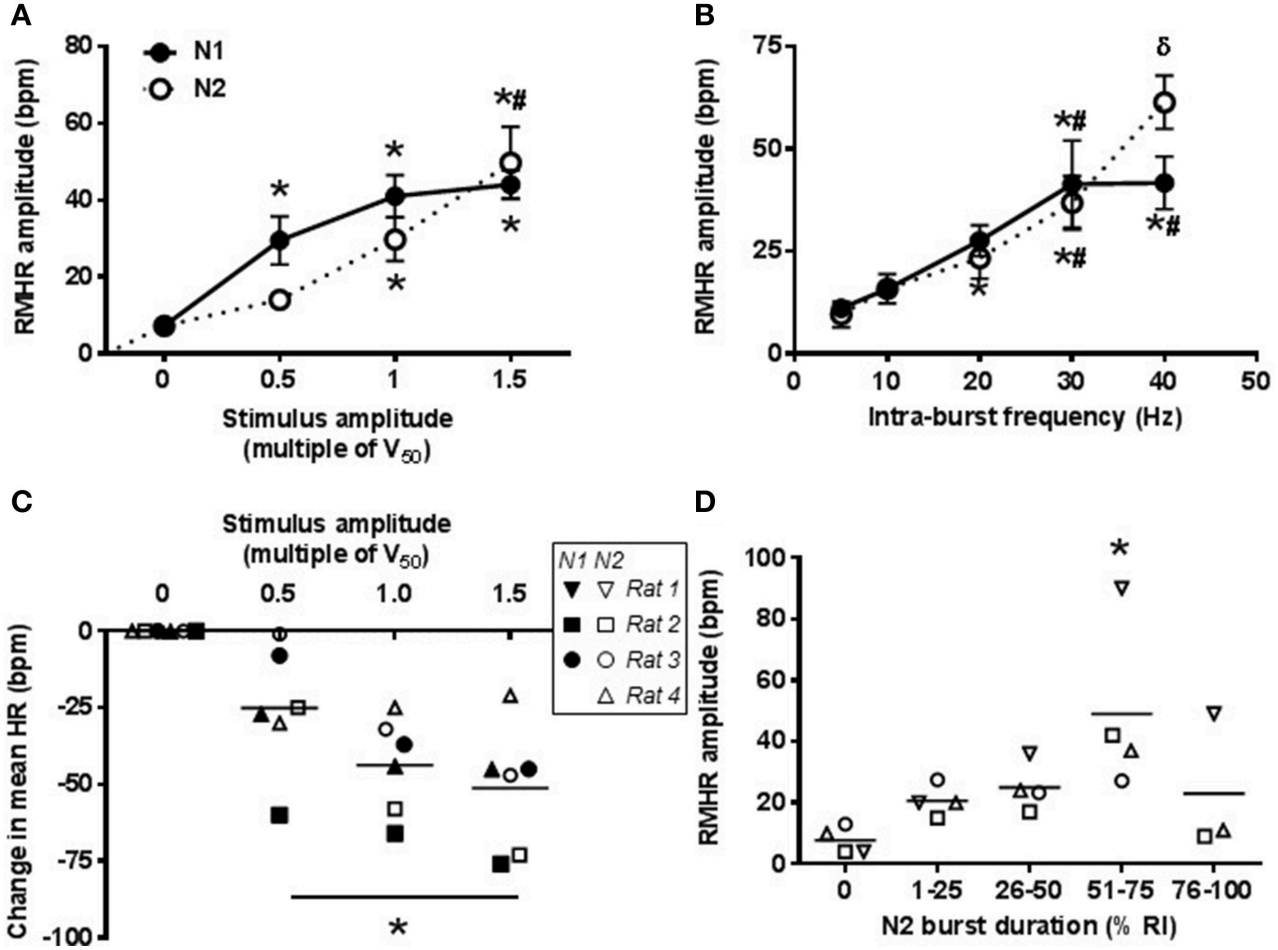
**RMHR generated by the VN-CPG: amplitude control**. The magnitude of RMHR amplitude during N1- and N2-mediated vagal nerve stimulation is linearly related to the stimulus amplitude **(A)** and intra-burst frequency **(B)**. A decrease in mean HR was observed during VN-CPG stimulus at all stimulus amplitudes (intra-burst frequency 20 Hz, burst duration 36% RI for N1 and N2, *n* = 3) **(C)**. N2 burst durations between 50 and 75% RI generated larger RMHR amplitudes compared to other burst durations and RSA amplitudes after unilateral vagotomy (0% RI) **(D)**. In panels **(C,D)**, the response from each animal is represented by a different symbol where filled symbols indicate a N1 response and open symbols indicate a N2 response. One-way ANOVA followed by student's *t*-test where ^*^P < 0.05 compared to baseline, ^#^*P* < 0.05 compared to lower frequency or amplitude of the same group and ^δ^*P* < 0.05 compared stimulus-matched N1. HR, heart rate; RI, respiratory interval; RMHR, respiratory-modulated heart rate; V_50_, voltage for half maximal HR response.

The amplitude of RMHR was controlled using the VN-CPG via adjustment of either intra-burst frequency or stimulus amplitude. The average voltage for half-maximal change in HR (V_50_) was 1.1 ± 0.1 V (*n* = 4). The stimulus amplitude was changed whilst maintaining 20 Hz intra-burst frequency (Figures 6A,C). N1 stimulus amplitude 0.5 × V_50_ significantly increased RMHR amplitude compared to the unstimulated HR waveform (*P* < 0.01) and further increases up to 1.5 × V_50_ caused incremental, but not statistically significant, increases in RMHR amplitude. Using N2 as the stimulus, RMHR amplitude began to increase from baseline at 1.0 × V_50_ (*P* = 0.02) and further increased at 1.5 × V_50_ (*P* = 0.01). The maximum RMHR amplitude (at 20 Hz intra-burst frequency) was of similar amplitude using either N1 (42 ± 6 bpm) or N2 (44 ± 10 bpm) outputs (Figure 6A). A decrease in mean HR (44 ± 8 bpm at 1.0 × V_50_, *n* = 3) was observed during vagal nerve stimulation using N1 and N2. Individual data points are shown in Figure 6C as the mean change in HR was variable between rats, but not between N1 and N2.

RMHR amplitude was also responsive to changes in intra-burst frequency (Figure 6B) and reached a plateau at 30 Hz using N1 of the VN-CPG (42 ± 5 bpm) whereas N2 of the VN-CPG produced a larger RMHR amplitude at 40 Hz intra-burst frequency (61 ± 5 bpm) (*P* < 0.05 compared to 30 Hz intra-burst frequency of N2).

Increasing N2 burst duration had a significant effect on RMHR amplitude when the burst duration was 51–75% of RI and RMHR amplitude decreased, on average, to unstimulated levels at burst durations above 76% (Figure 6D). Mean HR did not change significantly with increasing N2 burst duration. Interestingly, doubling N1 burst duration (28 ± 2 to 56 ± 5% of RI) decreased mean resting HR from 372 ± 8 to 347 ± 7 bpm (*P* = 0.04, *n* = 3) but did not change the amplitude of RMHR (*P* = 0.07, *n* = 3).

### Amplitude of RMHR generated using the PM-CPG

**Figure 7:**
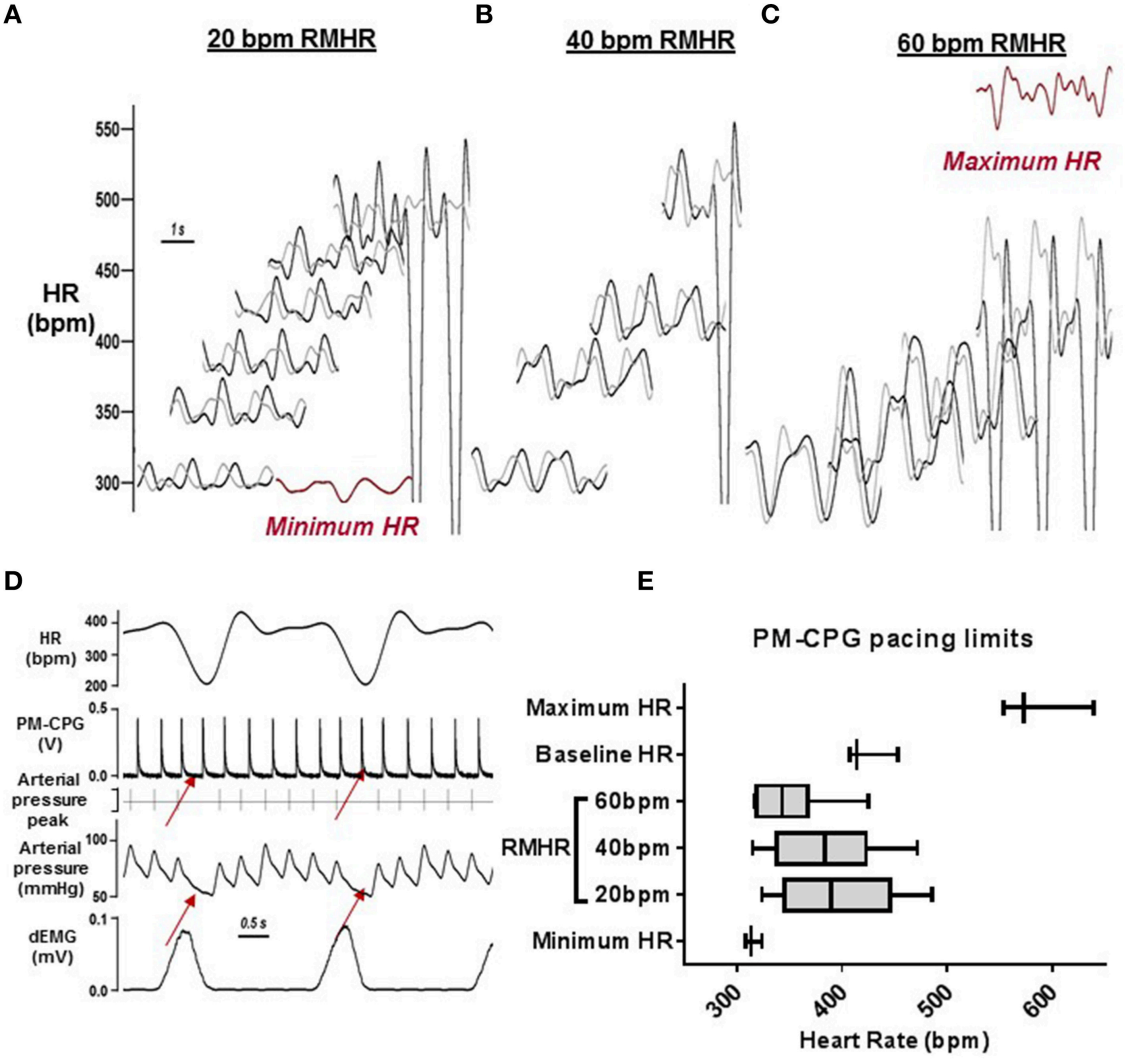
**RMHR generated by the PM-CPG: amplitude control. (A–C)** Representative traces of the range of RMHR amplitudes (20, 40, and 60 bpm) and mean HR imposed by the PM-CPG output. The minimum and maximum HR waveforms are in red. **(D)** A representative example of the arrhythmia occurrence that determined the upper limit of PM-CPG pacing (red arrows). **(E)** Average heart rates achieved during PM-CPG pacing (*n* = 3) to shows the physiological upper and lower limits of applying RMHR to rats, relative to their baseline, minimum (with β-adrenoceptor blockade), and maximum HR. dEMG, diaphragmatic electromyogram; HR, heart rate; PM-CPG, hCPG as cardiac pacemaker; RMHR, respiratory modulated heart rate.

The minimum HR during β-adrenergic receptor blockade was 310 ± 6 bpm and the maximum HR generated during tonic pacing was 574 ± 20 bpm (*n* = 4). Within these boundaries, rats were successfully paced using the PM-CPG at RMHR of amplitudes 20, 40, and 60 bpm from the minimum HR and upwards in 50 bpm incremental increases. Representative examples from one rat are shown in Figures 7A–C where the red trace is its minimum and maximum HR. Loss of pacing efficacy was observed in the arterial pressure trace and indicated the limits of RMHR pacing (representative example in Figure 7D). The minimum mean HR during 20, 40, and 60 bpm RMHR was 337 ± 8, 317 ± 2, and 300 ± 8 bpm respectively and the maximum mean HR was 463 ± 21, 479 ± 26, and 434 ± 31 bpm (Figure 7E). The HR could be effectively paced upwards from the minimum intrinsic HR with RMHR amplitudes up to 60 bpm. The HR during PM-CPG induced RMHR could not be paced up to the maximum HR generated during tonic pacing.

### Induction and phase control of respiratory modulation of heart rate using the PM-CPG

**Figure 8:**
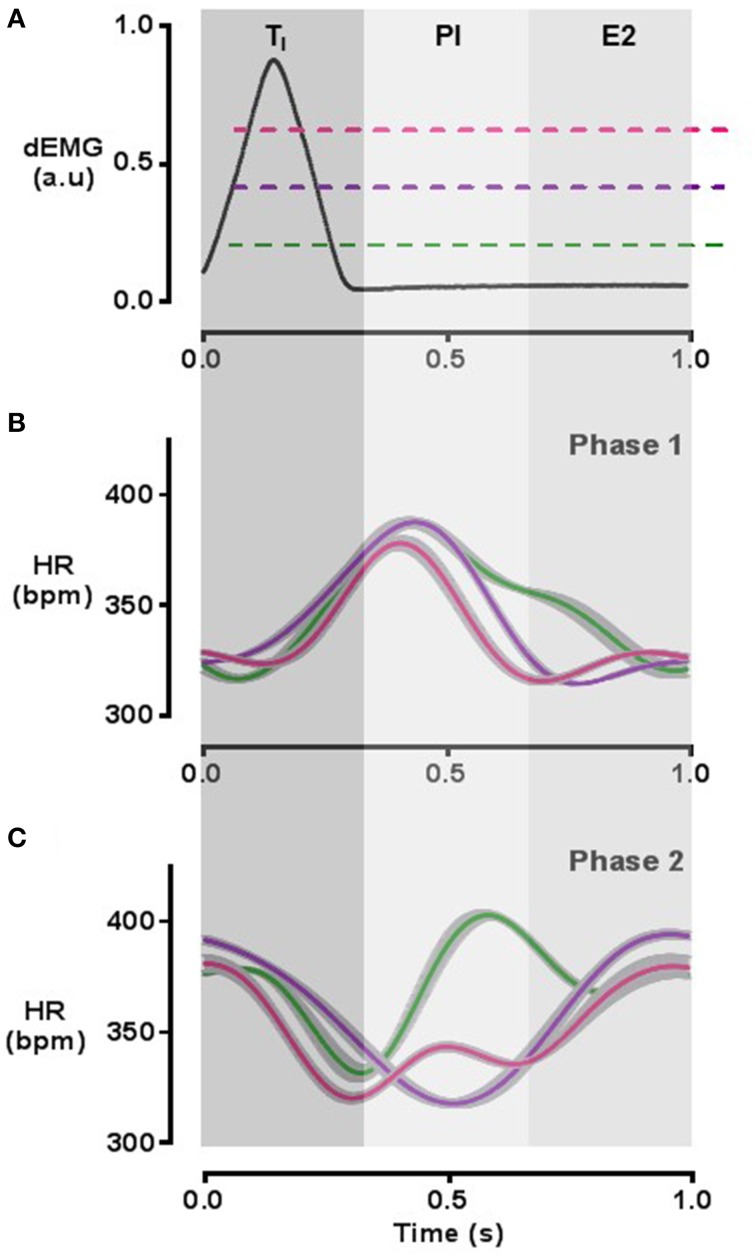
**RMHR generated by the PM-CPG: phase control**. The duration of high HR during Phase 1 PM-CPG pacing **(B)** and low HR during Phase 2 pacing **(C)** can be determined by adjusting the threshold at which the PM-CPG detects dEMG signal with respect to baseline noise **(A)**. HR waveforms are 60 s averages with standard error indicated in gray. The color of the waveform in each HR panel corresponds to the matching color of the approximated threshold (dashed lines) in the top panel. The respiratory cycle is divided into thirds as indicated by gray shading and the labels T_I_, PI, and E2. dEMG, diaphragmatic electromyogram; E2, late expiration; HR, heart rate; PI, post-inspiration; T_I_, period of inspiration.

Two distinct phases of RMHR could be generated by the PM-CPG, designated “Phase 1” and “Phase 2.” In Phase 1 mode, peak HR was synchronized to post-inspiration (PI) and trough HR began in the latter half of expiration (E2) and continued into inspiration (Figure 8B). In Phase 2 mode, trough HR was synchronized predominantly to PI and peak HR began in late expiration and continued into early inspiration (Figure 8C). The calculated phase shift of peak HR between Phase 1 and Phase 2 modes was 1.1 ± 0.2π radians (*n* = 3, *P* = 0.03), an inversion of the RMHR waveform. We found that the duration of the peak HR in Phase 1 mode could be adjusted by tuning the PM-CPG threshold at different amplitudes of the dEMG signal: Figure 8A is a representative dEMG burst with colored lines indicating 3 points in the continuum of possible thresholds that affect the width of peak HR during Phase 1 RMHR shown in Figure 8B. The duration of high HR could be tuned from the width of T_*I*_ (30% of RI, pink) up to 80% of RI (green). Changing the duration of high HR did not shift the phase of RMHR. In Phase 2, the dEMG threshold levels controlled the duration of trough HR to the same extent as Phase 1 RMHR pacing (Figure 8C).

### Comparison of the control of HR and RMHR by VN-CPG and PM-CPG during changes in respiratory rate

**Figure 9:**
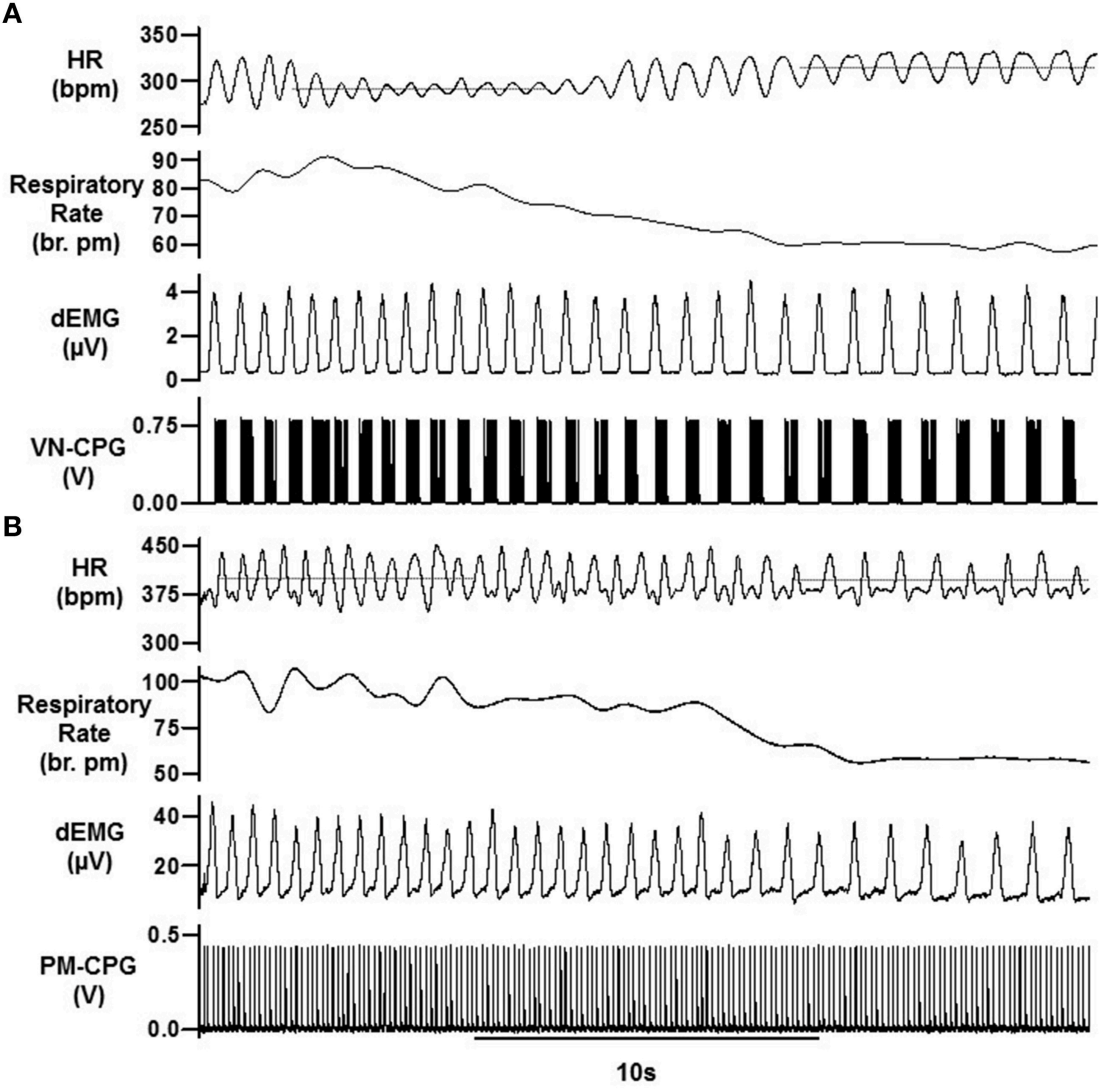
**Both VN- and PM-CPG maintain RMHR during changes in respiratory frequency**. Both the VN-CPG **(A)** and PM-CPG **(B)** maintain RMHR amplitude during changes in respiratory frequency although the amplitude is more variable using VN-CPG. The VN-CPG decreases mean HR (represented as dotted lines) whereas the PM-CPG maintains mean HR during increases in breathing rate. The heart rate trace is interpolated to fit a cubic spline instantaneous frequency of the electrocardiogram R-wave. dEMG, diaphragmatic electromyogram; HR, heart rate; PM-CPG, hCPG as cardiac pacemaker; VN-CPG, hCPG for vagal nerve stimulation.

The effect of changing respiratory rate on the HR during VN-CPG was tested using N1 (20 Hz intra-burst frequency, burst duration 30 ± 1% RI, *n* = 3). A decrease in respiratory rate from 89 ± 2 to 59 ± 2 breaths per min during VN-CPG control tended to increase mean HR (11 ± 3%, *n* = 3, *P* = 0.06). RMHR amplitude was typically larger at faster respiratory rates (80–86 breaths per minute). However, RMHR amplitude diminished when the respiratory rate reached 90 breaths per minute and remained low until the respiratory rate had decreased to ~72 breaths per minute (Figure 9A). The lack of direct control over RMHR amplitude and mean HR reflects a limitation to the degree of RMHR pacing using the VN-CPG.

RMHR amplitude (76 ± 7 bpm) generated using the PM-CPG was maintained during a 66 ± 3% change in respiratory rate (80 ± 9 to 56 ± 0 breaths per minute, *n* = 3, *P* = 0.40) and a representative example is shown in Figure 9B. Unlike VN-CPG pacing, mean HR did not change.

## Discussion

The aim of this study was to test two devices built from of a novel biofeedback hCPG platform for the application of modulating cardiac rate by respiratory frequency with a view to replicating intrinsic RSA. We have compared two different pacing methods to achieve this; the VN-CPG that initiated bradycardia via vagal nerve stimulation at different periods of the respiratory cycle and the PM-CPG that directly paced the heart at different rates during inspiration and expiration. The pattern of RMHR generated by N1 of the VN-CPG and the PM-CPG in Phase 2 were similar to each-other and most closely matched baseline RSA in our anesthetized rats. Both the VN-CPG and PM-CPG maintained RMHR during spontaneous changes in respiratory rate, highlighting the adaptive capacity of the novel devices. However, generating RMHR using the VN-CPG always decreased basal HR.

### Differences in RMHR generated by the VN-CPG and PM-CPG

The pattern of RMHR induced by the VN-CPG and PM-CPG could be adjusted to approach baseline RSA in anesthetized rats. In these experiments, we observed that intrinsic RSA showed a peak HR during mid-expiration and reached its nadir during early post-inspiration at baseline. The best approximation of this RSA was observed when using the PM-CPG in Phase 2 and N1 of the VN-CPG to induce RMHR (where N1 burst duration was 30–50% of the respiratory interval). The minimum HR per breath during RMHR induced using both of these approaches closely matched that of the intrinsic RSA generated by our anesthetized rats. However, peak HR during RMHR induced by the VN-CPG and the PM-CPG was shifted rightward compared to intrinsic RSA. Therefore, using N1 of the VN-CPG and the PM-CPG in Phase 2 was the best approximation to intrinsic RSA.

RSA is a manifestation of central respiratory gating of the cardiac vagal pre-ganglionic nerve (McAllen and Spyer, 1978; Gilbey et al., 1984) such that cardiac vagal nerve activity is dampened during inspiration and elevated post-inspiration (McAllen et al., 2011). This gating, in part, drives the respiratory modulation of HR observed in RSA, where HR accelerates during inspiration and decelerates during expiration (Hirsch and Bishop, 1981; Hayano et al., 1996; Eckberg, 2003; Bouairi et al., 2004). Inconsistencies in RSA pattern are dependent on species [cat (Gilbey et al., 1984), dog (Anrep et al., 1936), human (Koepchen et al., 1961; Eckberg et al., 1980)] and anesthesia (Bouairi et al., 2004). In this study in anesthetized rats, we consistently observed a reversal of the conventional RSA phase at baseline. This is not unprecedented as in isofluorane-anesthetized rats a similar pattern was observed (Tzeng et al., 2005) and might reflect an intrinsic cardiac mechanoreceptor response to increased intrathoracic pressure (Pathak, 1973; Taha et al., 1995).

The variation in RSA raises the question of whether the phase and pattern of RMHR are physiologically important. Although our current understanding of the physiological function of RSA is incomplete, there is some evidence to suggest phase is important. Hayano et al., applied artificial RSA and inverse RSA in anesthetized dogs and demonstrated that conventional RSA increased pulmonary blood perfusion when the ratio of dead space to total lung volume was lowest (i.e., during inspiration), optimizing pulmonary gas exchange. The opposite was observed during inverse RSA such that gas exchange was less efficient compared to unmodulated HR (Hayano et al., 1996). Elstad et al., demonstrated that RSA effectively shunts blood between the pulmonary artery and aorta such that the increase in HR is most effective during inspiration when venous return is highest (Elstad, 2012). In addition, mathematical modeling of a simplified cardiorespiratory system suggests not only that conventional phase RSA decreases myocardial energy demand, but inverse RSA increases myocardial energy demand compared to unmodulated HR (Ben-Tal et al., 2012). Therefore, we suggest that the phase of RMHR generated by our hCPG is physiologically important.

It is well known that the amplitude of RSA fluctuates with changes in breathing rate and is highest at lower respiratory rates (Hirsch and Bishop, 1981). Recent mathematical modeling of the effect of changing RSA amplitude on myocardial energy consumption suggest that the optimal range for minimizing energy consumption would be 10–15 bpm in humans (Ben-Tal et al., 2014). In this paper we have demonstrated fine control over RMHR amplitude using both the VN-CPG and PM-CPG. The amplitude of RMHR was indirectly controlled by the VN-CPG by manipulating its output in three ways: stimulus amplitude, intra-burst frequency, and burst duration. Changes in stimulus amplitude will affect RMHR amplitude up to the voltage at which all nerve fibers are activated. The value of this voltage depends on the nerve contact and on the VN-CPG output, which is limited to 4 V. Whilst the VN-CPG is virtually unlimited in its output intra-burst frequency, the RMHR amplitude response to N1-based stimulus plateaued at 30 Hz intra-burst frequency. This is in accordance with the reported maximal firing frequency of cardiac vagal motor neurons (McAllen et al., 2011). Increasing burst duration above 75% of the respiratory interval decreased RMHR amplitude suggesting the HR recovery time was insufficient for significant HR acceleration to have an effect. Indeed, the decreases in mean HR observed during RMHR pacing indicate that even periodic vagal nerve stimulation reduces the efficacy of HR acceleration to return HR to intrinsic levels. In contrast, RMHR amplitude could be directly controlled when using the PM-CPG by setting the spike frequency independently during inspiration and expiration. Therefore, the PM-CPG could be a more attractive option for clinical translation as the heart is paced both up and down.

A fundamental difference between using the VN-CPG and PM-CPG is that the former is a device that slows the heart (paces down) whereas the latter can only pace above baseline HR. Accordingly, the RMHR waveform generated by either neuron of the VN-CPG always decreased mean HR. This was unavoidable and may not be appropriate in conditions where cardiac output needs to be preserved or increased, such as heart failure. However, physiological RSA is maximal during slow, deep breathing when resting mean HR is relatively low (60–80 bpm in humans) (Eckberg, 1983; De Meersman, 1992). RSA tends to decrease in situations such as exercise where respiratory rate and HR increase (Hatfield et al., 1998; Taylor et al., 2001). Therefore, using the PM-CPG to generate RMHR above intrinsic HR could be most useful at rest and may require the concurrent use of β-adrenergic receptor blockade to lower mean HR before generating RMHR.

### VN-CPG vs. PM-CPG: A translational perspective

It is well established that loss of HRV, of which RSA is a major component, is a prognostic indicator of cardiovascular diseases including heart failure (HF) (Algra et al., 1993; Bigger et al., 1995; La Rovere et al., 1998). Whilst we do not fully understand the physiological importance of RSA, recent studies utilizing mathematical modeling suggest that RSA could conserve energy whilst maintaining cardiac output, a feature that could be a valuable addition to heart failure treatment. This loss of RSA in heart failure is a direct result of decreased vagal tone (Bibevski and Dunlap, 1999) and recent clinical trials in HF patients have aimed to address this imbalance by direct vagal nerve stimulation (Hauptman et al., 2012; De Ferrari et al., 2014; Premchand et al., 2014). Whilst some of these studies have reported improvements in cardiac function and patient health (Premchand et al., 2014; Zannad et al., 2015) it is not clear whether the mechanism underlying these improvements are related to increased HRV.

In this study we transected the vagus nerve to prevent afferent nerve traffic which caused respiratory disturbances. This would not be feasible in a clinical setting unless methods were developed to restrict nerve stimulation of the efferent fibers exclusively, perhaps by incorporating anodal block stimulation. Electrode leads and configurations have been developed in animal models that successfully directed the propagation of vagus nerve evoked action potentials down efferent fibers (Jones et al., 1995; Anholt et al., 2011). Another potential drawback of the VN-CPG approach comes from evidence suggesting that post-ganglionic vagal nerve transmission is impaired in a canine model of heart failure (Bibevski and Dunlap, 1999), a situation that would prevent the use of a VN-CPG for generating RMHR if this were the case in humans. Since the PM-CPG circumvents these issues it may have a better outcome in clinical applications if cardiac vagal ganglionic transmission is depressed in humans.

Current pacemakers do not recapitulate the lost HRV as they lack sophisticated biofeedback capabilities integrating in real time. Some attempts to rectify this have been made by incorporating an accelerometer into the design to detect the patient's activity level and adjust HR accordingly (Garrigue et al., 2002). Whilst activity-based HR pacing improves HRV compared to fixed-rate pacing (Raj et al., 2004), we believe that the PM-CPG, integrating real-time respiratory activity, could provide a significant advancement in pacing technology that would be more efficient at controlling cardiac rate whilst simultaneously reinstating physiologically based variability on a breath by breath basis, something that is lost in monotonic pacing.

The hCPG electronics that underlies both the VN- and PM-CPG was designed using analog circuits that have significant benefits over their digital counterparts (Nogaret et al., 2015). The unique advantage of analog electronics is that it allows accurate real-time integration of complex physiological feedback of neurons by the Hodgkin-Huxley model. Using a circuit design based on artificial neurons is also advantageous because neurons have a nonlinear response which is essential to synchronize aperiodic biological rhythms such as respiration and heart rate. Analog circuits present minimal overhead as analog to digital conversion is unnecessary, minimizing power consumption, and this presents a significant advantage in terms of miniaturization potential and extending battery life.

## Conclusion

We have demonstrated the utility of a novel biofeedback device for changing heart rate with every respiratory cycle in spontaneously breathing, anesthetized rats via the vagus nerve (VN-CPG) and with some modification of the device, via right atrial pacing (PM-CPG). The amplitude and phase of respiratory modulation of HR could be finely controlled using both approaches. However, the vagus nerve was transected and mean HR unavoidably decreased using the VN-CPG and this may limit its clinical application.

## Author contributions

EO conducted the experiments, analyzed the data and prepared the manuscript. AC and LZ built and tested the hCPGs and assisted with tuning the devices during *in vivo* experiments. RL assisted in conducting the experiments and reviewed the manuscript. HS was a key advisor. AN designed the devices, guided testing, prepared the technical sections of the manuscript, and sourced funding. JP advised in experimental design and manuscript preparation and sourced funding for the project.

## Funding

This work was supported by the Elizabeth Blackwell Institute and the British Heart Foundation under New Horizons grant NH/14/1/30761. RL was supported by the Sao Paulo Research Foundation (FAPESP).

### Conflict of interest statement

The authors declare that the research was conducted in the absence of any commercial or financial relationships that could be construed as a potential conflict of interest.

## Abbreviations

Φ: phase shift
dEMG: diaphragmatic electromyogram
E2: period of late expiration
hCPG: hardware central pattern generator
HR: heart rate
N1: neuron 1
N2: neuron 2
PI: period of post-inspiration
PM-CPG: hCPG format for use as a cardiac pacemaker
RI: respiratory interval
RMHR: respiratory modulated heart rate
RSA: respiratory sinus arrhythmia
T_I_: period of inspiration
V_50_: voltage for half maximal HR response
VN-CPG: hCPG for vagal nerve stimulation.
